# Bibliometric Analysis of Bronchopulmonary Dysplasia in Extremely Premature Infants in the Web of Science Database Using CiteSpace Software

**DOI:** 10.3389/fped.2021.705033

**Published:** 2021-08-20

**Authors:** Qin Zhou, Hai-Bo Kong, Bao-Mei He, Su-Ya Zhou

**Affiliations:** ^1^Department of Pediatrics, Zhejiang Provincial People's Hospital, People's Hospital of Hangzhou Medical College, Hangzhou, China; ^2^Department of Neonatology, Hangzhou Children's Hospital, Hangzhou, China

**Keywords:** bronchopulmonary dysplasia, extremely pre-mature infants, bibliometrics, CiteSpace, Web of Science

## Abstract

**Objectives:** To review the literature related to bronchopulmonary dysplasia in extremely pre-mature infants, summarize research direction, and report trends.

**Methods:** CiteSpace is a Java application which supports visual exploration with knowledge discovery in bibliographic databases. Relevant articles from 2008 to 2020 were retrieved from the Web of Science Core Collection database, and we extracted the following data: title, abstract, year, keywords, author, organization, journal and cited literature. We downloaded the data into CiteSpace (version 5.7.R3) to summarize countries, institutions, journals, and authors. We visualized the data with a knowledge map, collaborative network analysis, cluster analysis, and burst keyword analysis.

**Results:** We identified 610 articles on bronchopulmonary dysplasia in extremely pre-mature infants. The United States had the most articles on this topic (302 articles), followed by Canada (49 articles) and Germany (44 articles). The top three institutions, high-yield journals, and authors were all from the United States. The most common keywords were neurodevelopmental disorders, active perinatal care, mechanical ventilation, inflammation, pulmonary hypertension, low-dose hydrocortisone, development, and patent ductus arteriosus.

**Conclusions:** This study illustrates the trends and frontiers in the study of bronchopulmonary dysplasia in extremely pre-mature infants. The current research direction is to identify the risk factors in developing bronchopulmonary dysplasia in extremely pre-mature infants.

## Introduction

With the improvement of neonatal treatment, the survival rate of extremely pre-mature infants has increased significantly. Consequently, the incidence of bronchopulmonary dysplasia (BPD) is also increasing. Studies have shown that the incidence of BPD in extremely pre-mature infants with a gestational age of 22–27 weeks is 45% (1). The mortality and complication rate of pre-term infants with BPD is significantly higher than that of general pre-term infants. The prevention and management of BPD has become a major challenge in the field of perinatal and neonatal medicine. We conducted a literature review to further understand the research direction of bronchopulmonary dysplasia in extremely pre-mature infants and guide targeted research.

Bibliometrics is a text mining and analysis method that summarizes and analyzes research papers and academic journals. CiteSpace (http://cluster.cis.drexel.edu/~cchen/citespace/download/) is a scientific software that is often used to detect and visualize current scientific knowledge, detect trends in the literature, and determine future research directions (2, 3). CiteSpace uses information from the Web of Science Core Collection (WOSCC) and other database networks (4). This method can evaluate the corresponding data around knowledge gap involving important information including authors, journals, research institutions and keywords, and has been widely used in many fields such as medicine, geology, and ecology (5–7).

As far as we know, bibliometric techniques have not been applied to the area of BPD in extremely pre-mature infants. In this study, we used CiteSpace software to visualize how literature is developing investigating BPD in extremely pre-mature infants. We discuss the research directions in this field.

## Methods

### Data Sources

We used CiteSpace 5.7.R3 for the bibliometrics and visualization analysis, which was designed to provide critical information of the current knowledge for bronchopulmonary dysplasia in extremely pre-mature infants, including the fast-growth topical areas and citation hotspots. The articles were identified from a literature search using the WOSCC.

### Search Parameters

Our retrieval used the following Core Collection of Web of Science (WOS): SCI-EXPANDED, SSCI, A&HCI, CPCI-S, CPCI-SSH, BKCI-S, BKCI-SSH, ESCI, CCR-EXPANDED, and IC. We performed a Boolean search on two lists with the operator “and.” The first list included the following search structure: TS = (Infant, Extremely Pre-mature^*^ OR Extremely Pre-mature Infant^*^ OR Infants, Extremely Pre-mature^*^ OR Pre-mature Infant, Extremely^*^ OR Pre-mature Infants, Extremely^*^ OR Extremely Pre-term Infants^*^ OR Extremely Pre-term Infant^*^ OR Infant, Extremely Pre-term^*^ OR Infants, Extremely Pre-term^*^ OR Pre-term Infant, Extremely^*^ OR Pre-term Infants, Extremely^*^ OR Extremely Pre-mature Infants^*^). The second list included the following search structure: TS = (“Bronchopulmonary Dysplasia” OR “Dysplasia, Bronchopulmonary”). We searched articles (Document Type) in English (Languages) published between 2008 and 2020 (Retrieval time; Retrieval deadline: 2020.12.31).

### Data Export

This retrieved articles, which were then exported using the “other file format,” which we defined as “plain text.” The exported record content was “full records and cited references.”

### Analysis

We used the CiteSpace software to identify the citation bursts for the publication year, country/region, research institution, journal, author, research hotspot, and keywords.

This information was then presented as a knowledge graph using the following parameters: a 13-year period and a time slice of 1 year. The Term Sources were “Title, Abstract, Author Keywords (DE), and Keywords Plus (ID).” We set the Links parameters are “Strength: Cosine,” “Scope: Within Slices.”

In the network graph, different nodes represented various elements such as authors, institutions, countries, and keywords. The size of the nodes reflects the number or frequency of publications; the larger the node, the higher the number or frequency of publications. The connection lines between the nodes reflect the relationship between the co-operation or co-citation, the different colors within the nodes represent different times, and the color of the line reflects the years when the co-operation or co-citation first appeared (8). In addition, centrality reflects the role of the nodes in the knowledge network, and indicates the influence of one node on the other nodes. Nodes with greater centrality are more likely to become the key nodes in the network (9).

## Results

### Publication Years

**Figure 1:**
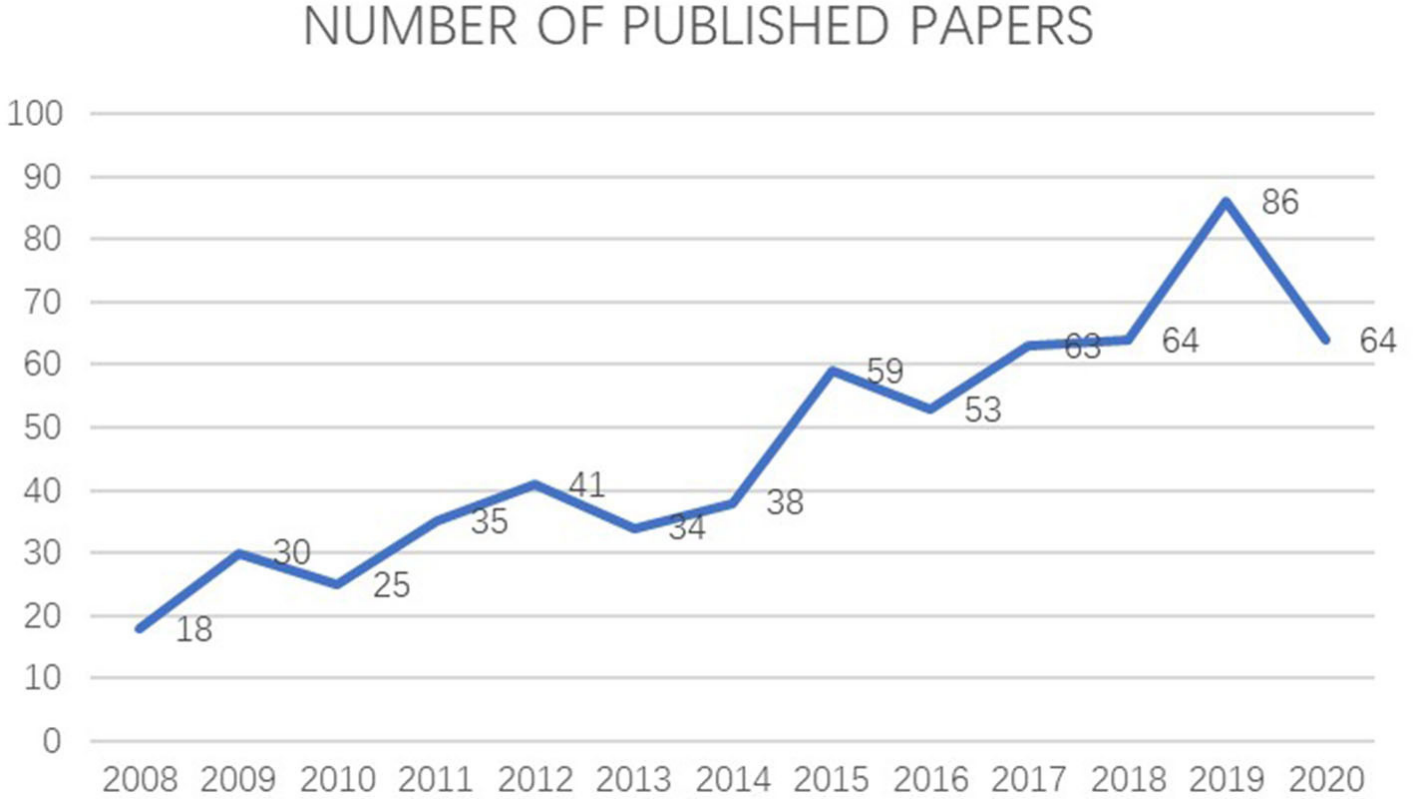
Trend chart of the number of articles published on BPD in extremely pre-mature infants.

We retrieved 610 documents related to BPD in extremely pre-mature infants. As shown in Figure 1, the number of related research articles on this topic is proliferating. The number of articles published increased from 18 in 2008 to 64 in 2020, showing that this research continues to develop and is one of the current hotspots in pediatrics (Figure 1).

### Countries and Institutions Analysis

**Figure 2:**
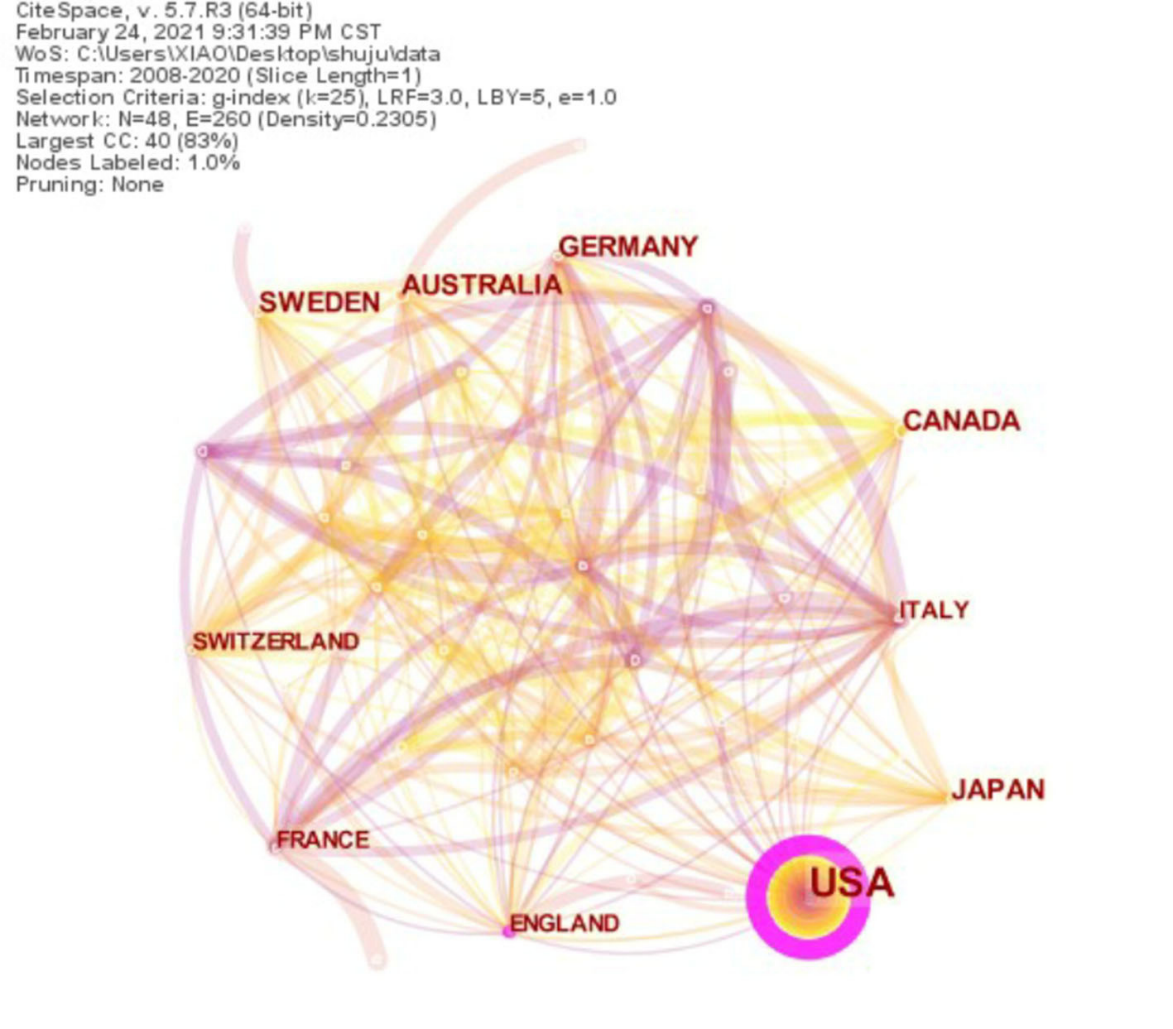
Top 10 collaboration network of country on BPD in extremely pre-mature infants.

We analyzed each country's cooperation network map for this topic (Figure 2) and used the Web of Science retrieval results to get the top 10 countries by the number of publications (Table 1). The United States has the most significant number of articles, followed by Canada, Germany, and Australia, showing that the countries mentioned above are in a leading position on this topic. The United States has a high national centrality (Act as an intermediary in the partnership) and plays an intermediary role in the national cooperation network. In other countries, centrality is relatively low, which shows that most institutions have a low influence and cooperation.

**Table 1:** Top 10 countries and institutes publishing research on BPD in extremely pre-mature infants.

**Country ranking**				**Institutional ranking**		
**Rank**	**Country**	**Publications**	**Centrality**	**Rank**	**Institution**	**Publications**
1	USA	302	0.42	1	Univ Alabama Birmingham	46
2	Canada	49	0.04	2	Case Western Reserve Univ	35
3	Germany	44	0.09	3	RTI Int	34
4	Australia	39	0.09	4	Brown Univ	32
5	Sweden	34	0.08	5	Emory Univ	32
6	Japan	34	0.03	6	Duke Univ	31
7	England	32	0.18	7	Wayne State Univ E	30
8	Italy	30	0.03	8	Eunice Kennedy Shriver Natl Inst Child Hlth and Hum	29
9	Switzerland	28	0.01	9	Univ Penn	26
10	France	24	0.08	10	Stanford Univ	25

**Figure 3:**
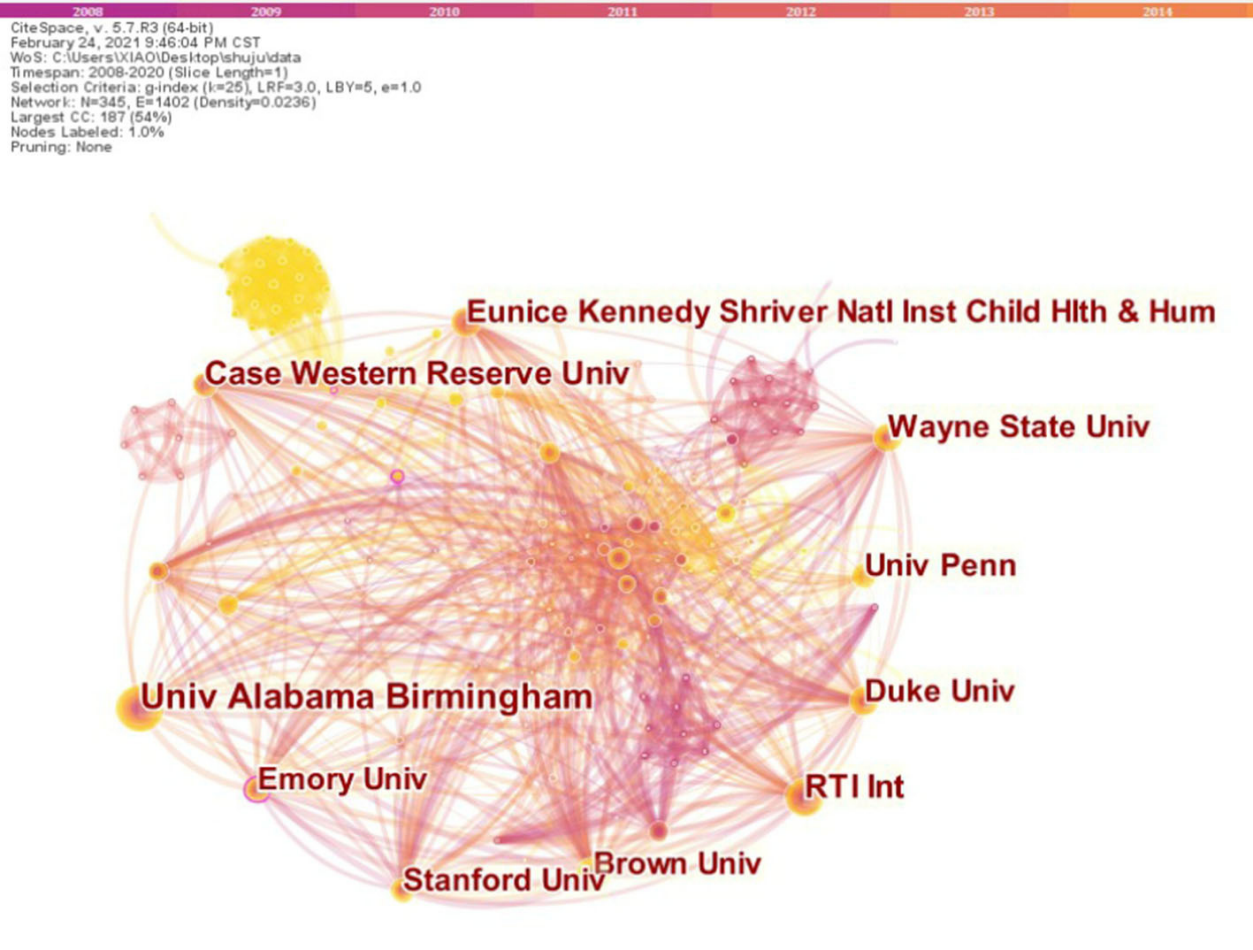
Top 10 collaboration network of institution on BPD in extremely pre-mature infants.

The top 5 institutions accounted for 320 articles (52.46%, Table 1). The top 5 research institutions are all in the United States: University of Alabama Birmingham, Case Western Reserve University, RTI International, Brown University, and Emory University. This finding shows that American research institutions are vital in the research field of BPD in extremely pre-mature infants, and they have carried out more in-depth and lasting research on the project with fruitful results. These research institutions have a dense network of cooperation, and they have more collaboration with other institutions (Figure 3).

### Journal and Author Analysis

Table 2 lists the top 10 journals by the number of publications. Among these journals, American journals account for five, and the remaining five are from European countries. The top 10 authors of the number of articles published in this field are all Americans (Table 3), indicating the significant research in this field is in the United States.

**Table 2:** Top 10 journals and published articles on BPD in extremely pre-mature infants.

**Rank**	**Journal**	**Publications**	**IF (2020)**	**Country**
1	The Journal of Pediatrics	52	3.739	USA
2	Journal of Perinatology	37	1.581	USA
3	Pediatrics	34	5.401	USA
4	American Journal of Perinatology	28	1.581	USA
5	Acta Paediatrica	27	2.265	Denmark
6	Neonatology	26	2.554	Switzerland
7	Pediatric Pulmonology	22	2.801	USA
8	Early Human Development	20	1.853	Netherlands
9	Archives of Disease in Childhood. Fetal and Neonatal Edition	17	3.776	England
10	Journal of Maternal Fetal Neonatal Medicine	10	1.812	England

**Table 3:** Top 10 authors with most publications on of BPD in extremely pre-mature infants.

**Rank**	**Author**	**Publications**
1	Abhik Das	34
2	Rosemary D. Higgins	30
3	Waldemar A. Carlo	30
4	NamasivayamAmbalavanan	26
5	Barbara J. Stoll	21
6	Abbot R. Laptook	19
7	Michele C. Walsh	17
8	Richard A. Ehrenkranz	13
9	Seetha Shankaran	13
10	T. Michael O'Shea	13

### Cited Literature and Author Analysis

**Figure 4:**
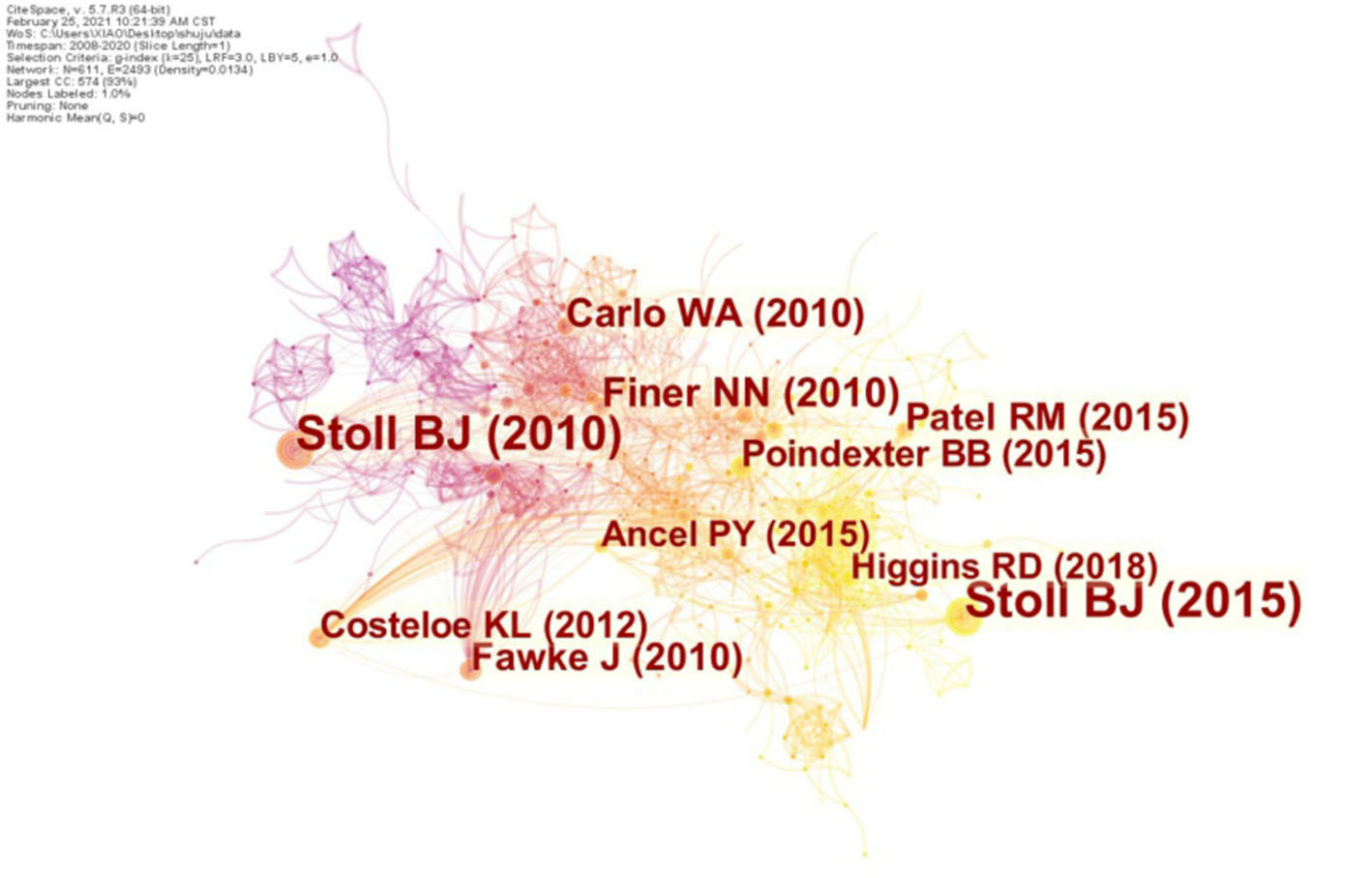
Top 10 highly cited articles on BPD in extremely pre-mature infants.

Table 4 and Figure 4 lists the most cited documents in this field. Among these documents, the author, Barbara Stoll (Emory University, United States), has the top two papers in terms of the number of citations in this area (Table 4). It shows that she has made outstanding contributions in this research direction. Most of the top five citations are published in the TOP journals: American Journal of Respiratory and Critical Care Medicine [Impact factor (IF) = 21.405], JAMA - Journal of the American Medical Association (IF = 56.272), New England Journal of Medicine (IF = 91.245), and Pediatrics (IF = 7.124). This pattern shows that the research results in BPD in extremely pre-mature infants are of great significance.

**Table 4:** Top 5 high cited articles related to BPD in extremely pre-mature infants.

**Rank**	**Author**	**Highly cited articles**	**Frequency**
1	Stoll B. J.	Trends in Care Practices, Morbidity, and Mortality of Extremely Preterm Neonates, 1993-2012 (JAMA-J Am Med Assoc, 2015)	64
2	Stoll B. J.	Neonatal Outcomes of Extremely Preterm Infants from the NICHD Neonatal Research Network (Pediatrics, 2010)	52
3	Finer N. N.	Early CPAP versus Surfactant in Extremely Preterm Infants (New Engl J Med, 2010)	26
4	Carlo W. A.	Target Ranges of Oxygen Saturation in Extremely Preterm Infants (New Engl J Med, 2010)	24
5	Fawke J.	Lung Function and Respiratory Symptoms at 11 Years in Children Born Extremely Preterm (Am J Resp Crit Care, 2010)	23

### High-Frequency Keywords Analysis

**Figure 5:**
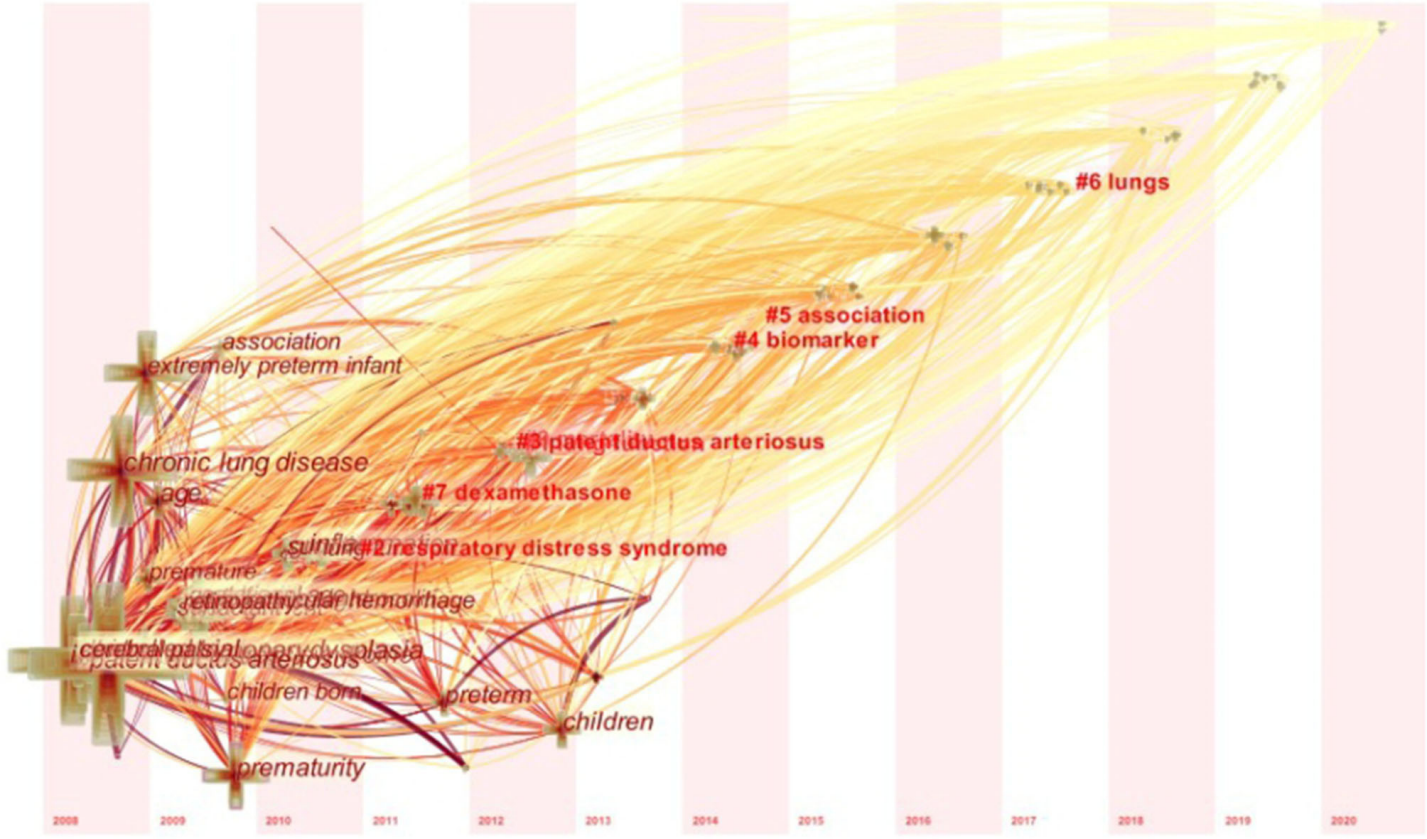
Timeline-zone of Keywords on BPD in extremely pre-mature infants.

The differences in high-frequency keywords at different periods show that the focus of research on BPD in extremely pre-mature infants has changed over the years. Before 2016, a significant number of articles on BPD in extremely pre-mature infants looked at an association with intrauterine infections. After 2016, the literature is more focused on treating BPD in extremely pre-mature infants and has increased significantly. Research on therapeutic drugs shifted from early dexamethasone to low-dose hydrocortisone. Caffein, inhaled nitric oxide, mesenchymal stem cells, vitamin D deficiency and supplementation, and others (Table 5), have also become new high-frequency keywords (Figure 5).

**Table 5:** High-frequency keywords of BPD in extremely pre-mature infants.

**Rank**	**Year**	**High-frequency keywords**
1	2008–2010	Low birth weight, respiratory distress syndrome, gestational age, chorioamnionitis, early adrenal insufficiency, endothelial growth factor, surfactant, continuous positive airway pressure, hypercapnia, pulmonary inflammation, dexamethasone, ventricle Bleeding
2	2011–2015	Low birth weight, respiratory distress syndrome, risk factors, cerebral palsy, necrotizing enterocolitis, chorioamnionitis, retinopathy, inflammation, surfactants, pulmonary hypertension, high-frequency oscillatory ventilation, dexamethasone therapy, inhaled monoxide nitrogen
3	2016–2020	Low birth weight, patent ductus arteriosus, treatment, risk factors, pulmonary hypertension, surfactants, respiratory distress syndrome, prevention, mechanical ventilation, inflammation, retinopathy, low-dose hydrocortisone, intraventricular hemorrhage, neurodevelopmental abnormalities, Active perinatal care, nutrition, necrotizing enterocolitis, chorioamnionitis, caffeine therapy, inhaled nitric oxide, endothelial growth factor, mesenchymal stem cells, vitamin D deficiency and supplementation, early adrenal insufficiency

### Keywords With Citation Bursts

**Figure 6:**
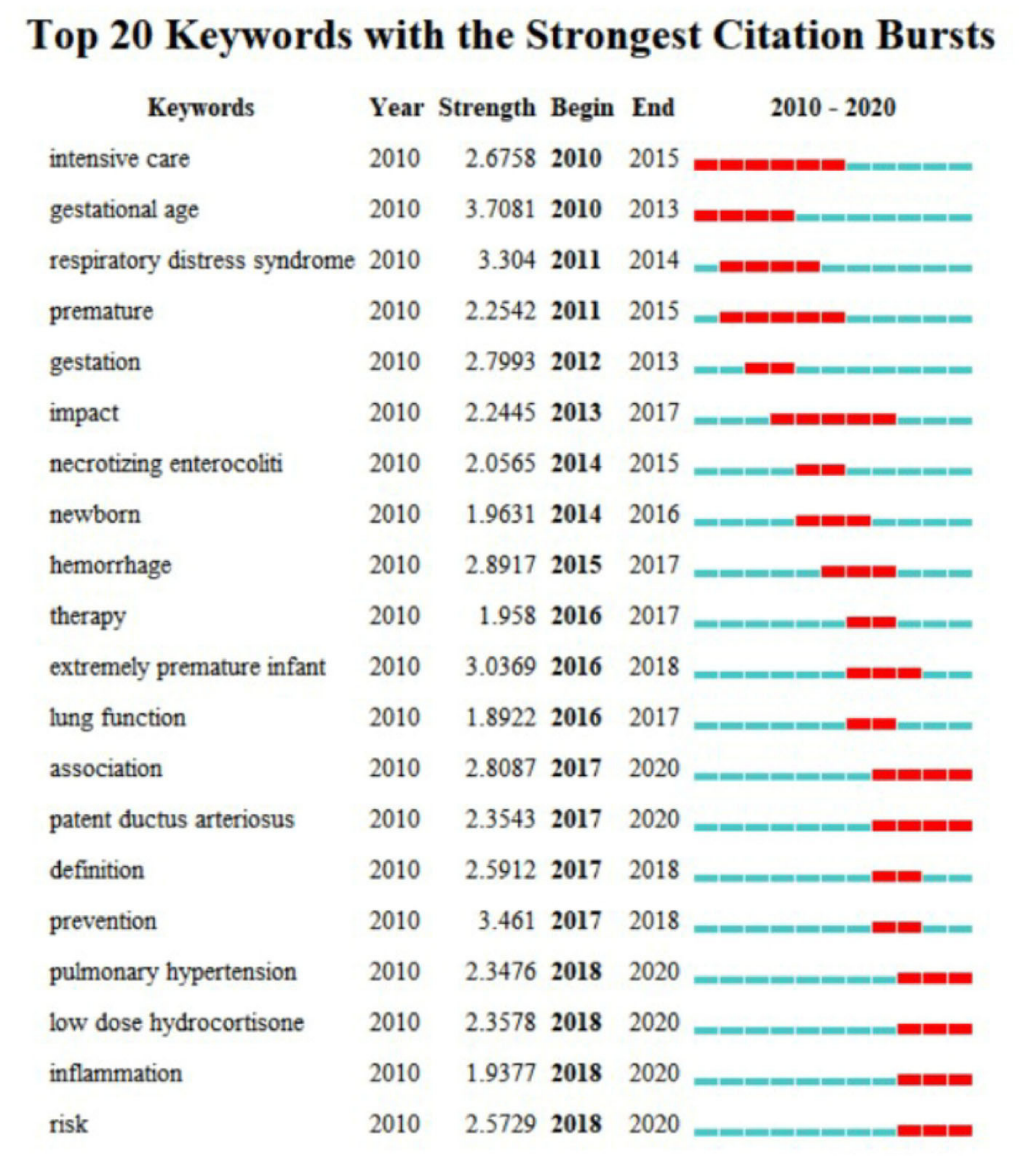
Burstness map of keywords on BPD in extremely pre-mature infants.

Figure 6 shows the top 20 keywords with the most substantial citation bursts. The blue line represents the time interval, while the red line shows the period in which a keyword had a burst. The keywords show the forefront of research on this topic. In the study of BPD in extremely pre-mature infants, the keywords with large mutation values that lasted until 2020 are highlighted: high-risk factors, inflammation, low-dose hydrocortisone, pulmonary hypertension, and patent ductus arteriosus. These may be the future trend of BPD research in extremely pre-mature infants.

## Discussion

We have analyzed the number of publications, countries, high-impact journals, and authors researching BPD in extremely pre-mature infants. We found that over the last 13 years, countries worldwide have increased research on this topic. The top 10 publications are all in developed countries, with the United States having the highest number of publications, academic institutions, high-impact journals, and authors.

The analysis of the results of both high-frequency keywords and keywords with citation bursts shows that the research focus of BPD in extremely pre-mature infants has changed over time. Since 2008, exploring the high-risk factors of BPD in extremely pre-mature infants has been a research hotspot that has determined that the occurrence of BPD is the result of multiple factors. Gestational age (GA), low birth weight, intrauterine infection, maternal chorioamnionitis, lung inflammation, and respiratory distress syndrome (RDS) are important factors in the pathogenesis of BPD. Since 2016, the interest in the relationship between patent ductus arteriosus (PDA) and BPD has gradually increased. Some research believed PDA with the hemodynamic effects due to the large left-to-right shunt, can cause pulmonary edema, which leads to prolonged mechanical ventilation time and increases the risk of developing BPD (10, 11). However, it is still not clear that interventions to close the PDA to reduce the risk of BPD in humans. Multiple studies suggest that the development of BPD is unchanged regardless of the PDA treatment strategy (12, 13).

Glucocorticoids have always been the most studied and, at the same time, the most controversial class of drugs for treating BPD in extremely pre-mature infants. Based on high-frequency keywords, the research on glucocorticoids has increased in the past 5 years. Currently, the short-term, low-dose dexamethasone regimen is still the most widely used (14). Short-term adverse effects include high blood pressure, hyperglycemia, gastrointestinal bleeding, and gastrointestinal perforation when using hormones in pre-mature infants. The hormones have adverse effects on neurodevelopment, including an increased risk of cerebral palsy (15). As a result, a high-dose long-term hormone to prevent BPD or as early treatment has been abandoned (16). Efficacy and safety of hormone therapy for bronchopulmonary dysplasia in extremely pre-mature infants need larger, multicenter, randomized, controlled trials.

In terms of other drug treatments for BPD in extremely pre-mature infants, research into caffeine and mesenchymal stem cell treatment has increased significantly in recent years. Most scholars believe that early use of caffeine is beneficial as a treatment of BPD (17, 18). Caffeine use within 48 h of birth can allow the removal of the ventilator earlier and reduce the incidence of BPD, PDA, and neurodevelopmental deficits (19). However, a randomized, placebo-controlled trial showed that early initiation of caffeine in pre-term infants did not reduce the age of first successful extubation. The finding suggest caution with early use of caffeine in mechanically ventilated pre-term infants until more efficacy and safety data become available (20). Therefore, more research is needed to clarify the starting time and duration of caffeine treatment.

In the past 5 years, several trials have investigated the treatment of BPD with cell therapy. Mesenchymal stem cells (MSCs) derived from human cord blood have received particular attention due to their easy isolation, low immunogenicity, and anti-inflammatory and repair properties. Four countries, South Korea (21), the United States (22), Spain (23), and China (24), have successfully carried out phase I or II clinical trials of MSCs to treat BPD. Published results show that intravenous or intratracheal injection of MSCs can change the composition of inflammatory factors in airway secretions, reduce lung inflammation and fibrosis, and reduce the severity of BPD; to date, no adverse reactions have been found (25). However, the mechanism of MSCs in repairing BPD is unclear. Therefore, the study of the pathophysiology of BPD, the use of MSCs for treatment, and transforming cell therapy into clinical use will be important areas of future research.

Research hotspots in the past 5 years have shown that research has gradually moved toward the complications and prognosis of BPD in extremely pre-mature infants. Neurodevelopmental disorders and pulmonary hypertension (PH) are the two major complications of BPD. The long-term neurodevelopmental outcome of pre-term infants with BPD is significantly higher than that of non-BPD pre-term infants (26). Neurodevelopment disorders risk factors include infections, poor growth, birth hypoxia, and later steroid use. PH is the most severe complication of BPD (27), with an incidence rate of 14–50% (28). This complication can progress to pulmonary heart disease (PHD) and significantly affect long-term prognosis. BPD-related PH has a mortality rate of up to 40% before 2 years of age (29).

## Strengths and Limitations

To our knowledge, this is the first study to use the co-occurrence and co-citation analysis methods by CiteSpace to perform bibliometric analysis and visual display of bronchopulmonary dysplasia in extremely pre-mature infants.

Our study does have limitations. We analyzed only English studies in the WOS due to software limitations. Therefore, the data may not be comprehensive, and our results may not be applicable to research published in other languages. Synonyms, when clustering keywords, can cause some overlaps between different categories of contents.

## Conclusions

We can determine the research status of BPD treatments and pathophysiology using CiteSpace. At present, the research hotspots of BPD tend to be the rational use of hormones, cell therapy, early detection, and follow-up of complications. Understanding the pathogenesis and treatment prospects of BPD in the future requires continuously discovering and solving related problems.

## Author Contributions

QZ and S-YZ designed the research subject. H-BK and QZ conducted literature retrieval and screening. B-MH and H-BK provided guidance in statistical analysis. QZ wrote the manuscript. S-YZ critically revised the manuscript. All authors read and approved the final manuscript.

## Conflict of Interest

The authors declare that the research was conducted in the absence of any commercial or financial relationships that could be construed as a potential conflict of interest.

## Publisher's Note

All claims expressed in this article are solely those of the authors and do not necessarily represent those of their affiliated organizations, or those of the publisher, the editors and the reviewers. Any product that may be evaluated in this article, or claim that may be made by its manufacturer, is not guaranteed or endorsed by the publisher.

## References

[1] Stoll BJ Hansen NI Bell EF Walsh MC Carlo WA Shankaran S. Trends in care practices, morbidity, and mortality of extremely preterm neonates, 1993-2012. JAMA. (2015) 314:1039–51. 10.1001/jama.2015.10244 26348753 PMC4787615

[2] Yao L Hui L Yang Z Chen X Xiao A. Freshwater microplastics pollution: detecting and visualizing emerging trends based on Citespace II. Chemosphere. (2020) 245:125627. 10.1016/j.chemosphere.2019.125627 31864046

[3] Chen C. Searching for intellectual turning points: progressive knowledge domain visualization. Proc Natl Acad Sci USA. (2004) 101(Suppl. 1):5303–10. 10.1073/pnas.0307513100 14724295 PMC387312

[4] Gao Y Shi S Ma W Chen J Cai Y Ge L. Bibliometric analysis of global research on PD-1 and PD-L1 in the field of cancer. Int Immunopharmacol. (2019) 72:374–84. 10.1016/j.intimp.2019.03.045 31030093

[5] Mund M Kloft B Bundschuh M Klingelhoefer D Groneberg DA Gerber A. Global research on smoking and pregnancy-a scientometric and gender analysis. Int J Environ Res Public Health. (2014) 11:5792–806. 10.3390/ijerph110605792 24879489 PMC4078548

[6] Dufault B Klar N. The quality of modern cross-sectional ecologic studies: a bibliometric review. Am J Epidemiol. (2011) 174:1101–7. 10.1093/aje/kwr241 21940800

[7] Zheng M Fu HZ Ho YS. Research trends and hotspots related to ammonia oxidation based on bibliometric analysis. Environ Sci Pollut Res Int. (2017) 24:20409–21. 10.1007/s11356-017-9711-0 28707243

[8] Liang C Luo A Zhong Z. Knowledge mapping of medication literacy study: a visualized analysis using CiteSpace. SAGE Open Med. (2018) 6:2050312118800199. 10.1177/2050312118800199 30245817 PMC6144508

[9] Chen C Chen Y. Searching for clinical evidence in CiteSpace. AMIA Annu Symp Proc. (2005) 2005:121–5. PMC1560638 16779014

[10] El-Khuffash A James AT Corcoran JD Dicker P Franklin O Elsayed YN. A patent ductus arteriosus severity score predicts chronic lung disease or death before discharge. J Pediatr. (2015) 167:1354–61.e2. 10.1016/j.jpeds.2015.09.028 26474706

[11] Clyman RI Hills NK Liebowitz M Johng S. Relationship between duration of infant exposure to a moderate-to-large patent ductus arteriosus shunt and the risk of developing bronchopulmonary dysplasia or death before 36 weeks. Am J Perinatol. (2020) 37:216–23. 10.1055/s-0039-1697672 31600791 PMC9940607

[12] Willis KA Weems MF. Hemodynamically significant patent ductus arteriosus and the development of bronchopulmonary dysplasia. Congenit Heart Dis. (2019) 14:27–32. 10.1111/chd.12691 30343505

[13] Clyman RI Liebowitz M Kaempf J Erdeve O Bulbul A Hakansson S. PDA-TOLERATE trial: an exploratory randomized controlled trial of treatment of moderate-to-large patent ductus arteriosus at 1 week of age. J Pediatr. (2019) 205:41–8.e6. 10.1016/j.jpeds.2018.09.012 30340932 PMC6502709

[14] Doyle LW Davis PG Morley CJ McPhee A Carlin JB. Low-dose dexamethasone facilitates extubation among chronically ventilator-dependent infants: a multicenter, international, randomized, controlled trial. Pediatrics. (2006) 117:75–83. 10.1542/peds.2004-2843 16396863

[15] Doyle LW Cheong JL Ehrenkranz RA Halliday HL. Late (> 7 days) systemic postnatal corticosteroids for prevention of bronchopulmonary dysplasia in preterm infants. Cochrane Database Syst Rev. (2017) 10:CD001145. 10.1002/14651858.CD001145.pub4 29063594 PMC6485440

[16] Jefferies AL. Postnatal corticosteroids to treat or prevent chronic lung disease in preterm infants. Paediatr Child Health. (2012) 17:573–4. 10.1093/pch/17.10.573 24294068 PMC3549698

[17] Schmidt B Roberts RS Davis P Doyle LW Barrington KJ Ohlsson A. Caffeine therapy for apnea of prematurity. N Engl J Med. (2006) 354:2112–21. 10.1056/NEJMoa054065 16707748

[18] Schmidt B Roberts RS Davis P Doyle LW Barrington KJ Ohlsson A. Long-term effects of caffeine therapy for apnea of prematurity. N Engl J Med. (2007) 357:1893–902. 10.1056/NEJMoa073679 17989382

[19] Lodha A Seshia M McMillan DD Barrington K Yang J Lee SK. Association of early caffeine administration and neonatal outcomes in very preterm neonates. JAMA Pediatr. (2015) 169:33–8. 10.1001/jamapediatrics.2014.2223 25402629

[20] Amaro CM Bello JA Jain D Ramnath A D'Ugard C Vanbuskirk S. Early caffeine and weaning from mechanical ventilation in preterm infants: a randomized, placebo-controlled trial. J Pediatr. (2018) 196:52–7. 10.1016/j.jpeds.2018.01.010 29519541

[21] Ahn SY Chang YS Lee MH Sung SI Lee BS Kim KS. Stem cells for bronchopulmonary dysplasia in preterm infants: a randomized controlled phase II trial. Stem Cells Transl Med. (2021) 10:1129–37. 10.1002/sctm.20-0330 33876883 PMC8284779

[22] Powell SB Silvestri JM. Safety of intratracheal administration of human umbilical cord blood derived mesenchymal stromal cells in extremely low birth weight preterm infants. J Pediatr. (2019) 210:209–13.e2. 10.1016/j.jpeds.2019.02.029 30992220

[23] Mesenchymal Stem Cell Therapy for Bronchopulmonary Dysplasia in Preterm Babies. ClinicalTrials.gov Identifier: NCT02443961. (Active, not recruiting).

[24] Stem Cells for Bronchopulmonary Dysplasia. ClinicalTrials.gov Identifier: NCT03378063. (Recruiting).

[25] Namba F. Mesenchymal stem cells for the prevention of bronchopulmonary dysplasia. Pediatr Int. (2019) 61:945–50. 10.1111/ped.14001 31487104

[26] Smith VC Zupancic JA McCormick MC Croen LA Greene J Escobar GJ. Rehospitalization in the first year of life among infants with bronchopulmonary dysplasia. J Pediatr. (2004) 144:799–803. 10.1016/j.jpeds.2004.03.026 15192629

[27] Krishnan U Feinstein JA Adatia I Austin ED Mullen MP Hopper RK. Evaluation and management of pulmonary hypertension in children with bronchopulmonary dysplasia. J Pediatr. (2017) 188:24–34.e1. 10.1016/j.jpeds.2017.05.029 28645441

[28] Mourani PM Abman SH. Pulmonary hypertension and vascular abnormalities in bronchopulmonary dysplasia. Clin Perinatol. (2015) 42:839–55. 10.1016/j.clp.2015.08.010 26593082 PMC5863545

[29] Arjaans S Zwart EAH Ploegstra MJ Bos AF Kooi EMW Hillege HL. Identification of gaps in the current knowledge on pulmonary hypertension in extremely preterm infants: a systematic review and meta-analysis. Paediatr Perinat Epidemiol. (2018) 32:258–67. 10.1111/ppe.12444 29341209

